# To Treat or Not to Treat Subclinical Hypothyroidism, What Is the Evidence?

**DOI:** 10.3390/medicina56010040

**Published:** 2020-01-19

**Authors:** Jan Calissendorff, Henrik Falhammar

**Affiliations:** 1 Department of Molecular Medicine and Surgery, Karolinska Institutet, SE-171 76 Stockholm, Sweden; henrik.falhammar@ki.se; 2 Department of Endocrinology, Metabolism and Diabetes, Karolinska University Hospital, SE-171 76 Stockholm, Sweden

**Keywords:** subclinical hypothyroidism, levothyroxine, overtreatment

## Abstract

Objective: levothyroxine prescriptions have increased remarkably during the last decade, and it is most likely to be prescribed in subclinical hypothyroidism. The aim of this review was to present data on when levothyroxine treatment should be initiated, and the effects of treatment in subclinical hypothyroidism on symptoms such as weight, quality of life, vitality, cognition, and cardiovascular disease. We also discuss evidence for different thyroid-hormone medications. In addition, the option to withhold medication when there is uncertain diagnosis or lack of clinical improvement is discussed. Methods: a literature search in PubMed on the term “treatment of subclinical hypothyroidism” in combination with “quality of life”, “weight”, “cognition”, and “cerebrovascular disease”. Results: current research supports that levothyroxine should be initiated in patients with a thyroid stimulating hormone (TSH) >10 mIU/L. Treatment for hypothyroidism is becoming more frequent. Symptoms related to vitality, weight, and quality of life in subclinical disease often persist with levothyroxine treatment, and other causes should be explored. Patients with cardiovascular-risk factors may benefit from treatment, especially younger patients. Caution is necessary when treating elderly subjects with levothyroxine. Conclusion: lifelong treatment with levothyroxine should normally only be considered in manifest hypothyroidism. However, in subclinical hypothyroidism with a TSH >10 mIU/L, therapy is indicated. In milder subclinical forms, a wait-and-see strategy is advocated to see if normalization occurs. Subgroups with cardiovascular risk and subclinical hypothyroidism may benefit from levothyroxine therapy.

## 1. Introduction

Hypothyroidism is one of the most common endocrine disorders. Clinical symptoms vary, from mild unspecific symptoms such as tiredness, cold intolerance, lack of vitality, and obstipation to life-threatening myxedema. In myxedema, there is also increased sensitivity to pharmacotherapy, confusion, areflexia, megacolon, and the risk of death [1]. In primary hypothyroidism pituitary derived thyroid stimulating hormone (TSH), thyrotropin, is increased to more than 10 mIU/L, with a simultaneous decrease in free thyroxin (T4) [2]. A majority of patients also have measurable autoantibodies against thyroid peroxidase (TPO ab), a vital enzyme in thyroid-hormone synthesis, as a marker for autoimmune thyroid disease.

Subclinical hypothyroidism is characterized by increased TSH (5–10 mIU/L) and normal fT4. In more severe forms, normal fT4 is found in the face of TSH >10 mIU/L [3]. As the term “subclinical” implies, this is a laboratory diagnosis, and prevalence is estimated to 12%–18% [4,5]. However, the link to developing hypothyroid symptoms is uncertain. Progression to manifest hypothyroidism is around 4% per year in the presence of TPO ab, and 2% per year if not present [6]. Fifteen years ago, the effects on hypothyroid symptoms in mild disease with a TSH of 4.5–10 mIU/L were not demonstrated, and routine treatment was discouraged [7]. This was controversial, and it was claimed that there was no harm in treatment in these cases, since the aim was to normalize thyroid parameters [8]. Indication for treatment is according to most guidelines a TSH >10 mIU/L [4] (Table 1). The European Thyroid Association (ETA) guidelines favor treatment in more severe forms. In those with milder forms, treatment could be considered with levothyroxine in cases with repeated measures of TSH between 5–10 mIU/L, and symptoms compatible with hypothyroidism. If a symptomatic response is not reached 3–4 months after TSH normalization, treatment should be stopped [3]. This is invariably difficult, as hypothyroid symptoms are unspecific by nature, and the decision to treat or not has to be individualized [9]. Factors such as age, smoking, weight, and ethnicity should be considered. Black people and smokers tend to have lower TSH, and older persons and iodine sufficient populations have higher levels [10,11]. During the last decade, the medical benefits and expectations of clinical improvement with treatment have gained increased attention [2]. A recent review declared that most cases with subclinical hypothyroidism can be observed without initiating treatment [12]. A recent guideline even suggested that treatment should not be commenced before TSH was >20 mIU/L, although evidence for this was much weaker in patients younger than 65 years old [13]. Levothyroxine is now the most common medication in the United States (US), and the third most common in the United Kingdom (UK) [14,15]. In Sweden, prescriptions have increased by 48% between 2006 and 2018, and almost 5% of the population is treated with levothyroxine (www.socialstyrelsen.se/register/halsodataregister/lakemedelsregistret). In contrast to guidelines, median initial TSH level fell between 2001 and 2009 in the UK from 8.7 to 7.9 mIU/L in 52,298 patients started on levothyroxine treatment [16]. Thus, a declining threshold in TSH levels, before medication with thyroid hormones is initiated, is the most plausible explanation for increased levothyroxine prescriptions, as no data support an increased incidence of hypothyroidism. Increased testing of thyroid hormones has also occurred, so more patients with subclinical disease were found [17].

Before initiation of levothyroxine therapy in subclinical hypothyroidism, a repeated control of TSH level within 3 months is imperative. This is important, as a transient elevation of TSH levels that normalizes within 3 months has been reported in 60% of cases [21,22], and after 5 years in 62% of cases [23]. In other diseases, such as myocardial infarction, septicemia, influenza, and during thyroiditis, TSH can temporarily indicate hypothyroidism [24,25]. Transient TSH elevation is also common the first months after commencing amiodarone [26]. Falsely elevated TSH levels can be found in cases with macro-TSH, which can be analyzed by precipitation with polyethylene glycol. Macro-TSH was noted in 15 out of 1901 (0.79%) patients with subclinical hypothyroidism [27], and is more common in clinically euthyroid individuals with a TSH >10 mIU/L [28]. In instances of iodine excess, both low and elevated TSH can be found with increased peripheral hormones, indicating a higher risk for hypothyroidism in iodine-sufficient populations. Furthermore, heterophilic antibodies may cause asymmetric levels of fT4 and/or free triiodothyronine (fT3), and TSH elevation, which can be evaluated by simultaneously comparing the different thyroid hormones with different assays [29,30].

Treatment with levothyroxine should be commenced in subclinical hypothyroidism (TSH > 4.0 mIU/L) in females planning pregnancy and if found during ongoing pregnancy [31], since normal thyroid function decreases the risk of miscarriage and other pregnancy complications.

Both patients and prescribers can expect great alleviation of decreased functions, such as cognition, memory, vitality, depression, and symptoms such as weight gain, when treating subclinical hypothyroidism [32]. When this relief fails to occur, the question of cause arises [33]. A number of investigations have recently aimed to clarify this. Patients now often demand and expect alternative pharmacotherapy with liothyronine or capsules with thyroid extracts from pigs. These could potentially have an improved effect compared to that of synthetic levothyroxine. More investigation is needed to see if there is scientific support for such a notion.

The aim of this review is to present data on when levothyroxine treatment should be initiated in subclinical hypothyroidism, the effects of levothyroxine treatment on aspects such as weight, quality of life, vitality, and cognition in these patients. We also aimed to investigate support for treatment in patients with cardiovascular risks and to discuss the evidence for alternative medications. The possibilities of withholding medication when there is an uncertain diagnosis is briefly discussed.

## 2. Methods

In this review, we performed a literature search in PubMed on the term “treatment of subclinical hypothyroidism” in combination with “quality of life”, “weight”, “cognition”, and “cerebrovascular disease”. Articles relevant to these searches were also identified in the authors’ personal files.

## 3. Results

### 3.1. Is This a Patient With Hypothyroidism?

To diagnose manifest hypothyroidism is relatively easy with clinical evaluation and blood tests. Premature treatment with thyroid hormones without a manifest disease in the thyroid increased the risk for hyperthyroidism with symptoms such as tiredness, weight loss, and restlessness as well as increased cardiac risk, and atrial fibrillation above all [34]. Supraphysiological doses with levothyroxine aiming at suppressed TSH in thyroid-cancer patients were associated with increased risk of cardiovascular and all-cause mortality, hazard ratios 3.35 (95% CI, 1.66 to 6.74) and 4.40 (95% CI, 3.15 to 6.14), respectively [35]. Such overtreatment can be the result in patients with levothyroxine medication treated for symptoms like weight gain or tiredness. Ordering copies of initial blood tests can be of value in those circumstances. In untreated subjects a differential diagnostic approach is necessary as other conditions can go along with symptoms similar to hypothyroidism. If markers for autoimmune disease exist (e.g., TPO ab), this increases the risk also for other autoimmune diseases, such as rheumatoid arthritis, type 1 diabetes, pernicious anemia, celiac disease, and Addison’s disease [36]. The latter could also result in an increase in TSH levels [37]. Other somatic diseases such as anemia, heart failure, and malignancies, as well as social and psychiatric conditions must be considered. Fatigue syndrome could also evoke symptoms mimicking hypothyroidism. An insufficient diet in relation to energy requirements may also explain reduced physical and mental vitality. Thus, if medication is initiated in subclinical hypothyroidism, symptoms can persist, and a reassessment of the cause of the symptoms is necessary. Unspecific symptoms such as fatigue are very common in the general population, occurring in 11%–33% [38,39,40,41]. Moreover, up to 25% of the euthyroid population may suffer from hypothyroid similar symptoms [42].

### 3.2. Effect of Initiating Treatment in Subclinical Hypothyroidism

Biochemical findings of subclinical hypothyroidism can be found in asymptomatic persons. Such individuals who did not seek medical evaluation, had superior wellbeing than their euthyroid counterparts did, measured with a General Health Questionnaire and neuropsychological tests [43]. In a recent meta-analysis, there was no alleviation in quality of life, cognition, blood pressure, or body mass index (BMI) with levothyroxine therapy in subclinical hypothyroidism [44]. In a randomized trial of older individuals, there was no relief in hypothyroid symptoms or tiredness with pharmacotherapy [21]. The increased levothyroxine prescriptions could thus be questioned. It is reasonable to follow patients with subclinical hypothyroidism and evaluate other factors which may cause the symptoms. Furthermore, in a Greek study of patients with ongoing medication for hypothyroidism, where initial diagnosis was uncertain, treatment was re-evaluated [45]. In all, 291 patients (median age 48 years) had their treatment with levothyroxine withheld. After 6–8 weeks, 177 (61%) had normal thyroid-function tests, while the remainder were hypothyroid. There was no difference according to sex, age, BMI, levothyroxine dose, antibodies, or duration of treatment. Echogenicity, evaluated with thyroid ultrasound, was significantly lower in those with persistent hypothyroidism. Thus, diagnosis and substitution therapy could be doubted, and levothyroxine should be reconsidered in cases with uncertain diagnosis.

### 3.3. Still Decreased Well-Being After Initiating Treatment in Patient With Subclinical Hypothyroidism

The aim of treating hypothyroid patients is to relieve symptoms with levothyroxine by reaching reference intervals for TSH [1]. There are several studies describing decreased well-being in patients with hypothyroidism. In one study, the levothyroxine dose was titrated in 52 patients into 3 groups with normal TSH values (0.3 ± 0.1, 1.1 ± 0.2, and 2.8 ± 0.4 mIU/L, respectively). Patients were randomly assigned to all these groups for 8 weeks each, without any effect on quality of life, well-being, or hypothyroid symptoms [46]. In another study, impaired psychological well-being was found in patients with normal TSH levels during treatment [47]. Moreover, in a Dutch investigation, well-being was also decreased in levothyroxine-treated euthyroid subjects measured with Symptom Check List-90 total score and the 36-item Rand Health Survey subscales for “mental health” and “vitality” [48]. Patients also had poor performance in various domains of attention and verbal memory when neurocognition was evaluated. However, in neither of these 2 latter studies were initial TSH levels or comorbidities reported. This could imply uncertainty as to why these patients were treated with levothyroxine, but also that there could be imperfection in the current treatment in some individuals, as in other investigations, in 35%–60% of treated subjects the dose was either too low or too high [16,49]. Other diseases may also influence the results. New medications, mainly long-acting T3 [50], may in the future improve treatment for the subgroup of 5%–10% of hypothyroid patients treated with levothyroxine and normal TSH and fT4 levels found to have decreased psychological well-being, depression, or anxiety [51].

Synthetic T4, levothyroxine, has been the standard treatment for hypothyroidism for more than 40 years. A more even concentration can be acquired with levothyroxine than with older preparations of capsules containing thyroid hormones from pigs [52,53], so called desiccated thyroid extract (DTE). DTE contains more T3 than the human thyroid does (4.2:1 vs. 14:1) with a 1 grain (65 mg) DTE tablet containing 38 μg of T4 and 9 μg of T3. When synthetic levothyroxine became available, several small observational studies with 10–40 participants compared the 2 preparations [54,55,56]. All showed that the synthetic compound was superior, giving a more stable substitution dose and decreasing the risk of supraphysiological doses. A modern study showed comparable plasma levels of TSH and fT4 between blinded standard treatment and DTE [57]. The 78 included participants changed medication after 4 months. Neuropsychological investigation did not display any difference between the 2 medications. When the study ended, 49% of the patients claimed to prefer DTE, and they had also had modest weight loss. There are no longitudinal data or safety assessments of DTE usage. It is generally difficult to find the right DTE dose. It is unknown whether this is due to the high content of T3 or to the uneven concentration in different batches of capsules. In 2018, an online survey was presented where patients reported their satisfaction with their medication and comorbidities [58]. Of these, 43% had Hashimoto thyroiditis, and the ratio of women to men was 22:1. Of the participants, 6949 used levothyroxine, 978 a combination (levothyroxine + liothyronine), and 3239 DTE. Those with self-reported depression were excluded. The remaining patients stated how pleased they were with the treatment on a 10 graded scale, where 10 denoted 0 symptoms. Those on levothyroxine reported 5, those on a combination 6, and those on DTE 7. However, conclusions are difficult to make, as the selection is unclear, and it was also uncertain whether or not patients had adequate doses. Moreover, the participants were to an unusual extent less satisfied with their treatment, which sheds some light that there could be inadequate efficacy with the treatment, unknown comorbidity or other factors. Most likely, there was bias present, and this study cannot be used to reflect the general hypothyroid population.

A healthy thyroid gland synthesizes and secretes T4, but also the more biologically potent T3. The ratio of T3 to T4 is approximately 1:14 [1]. T3 is acquired mainly by peripheral deiodination, which also applies when T4 is taken orally as levothyroxine. Liothyronine (T3) has not been developed for long-term substitution for hypothyroidism, but has grown to become a complementary treatment to monotherapy with levothyroxine. There is some logic to this, since when endogenous T3 is not available in hypothyroidism, complete substitution could be achieved by adding liothyronine. In a study of thyroidectomized patients (*n* = 1811), all on levothyroxine substitution, fT4 was slightly higher, and fT3 lower (within the normal range), compared to the levels of euthyroid patients (*n* = 3875) without thyroid medication [59]. Whether this affects quality of life and/or hypothyroid symptoms is uncertain [1]. Several studies with combination therapy using T4 and T3 with different designs and varying relations between the dose of T4 and T3 have been presented in meta-analysis [60]. Whether quality of life, cognition, weight, memory, depression, and vitality differed between monotherapy and combination treatment were evaluated. Only 1 randomized trial (*n* = 59) showed superiority for the combination therapy in different scores for quality of life, depression, anxiety rating scales, and patient preference compared to standard treatment [61]. Weight decreased by 1.5 kg in the combination-treated patients. All other studies found neutral effects when comparing factors such as cognition, memory, and quality of life [62]. In the included studies, the cause of hypothyroidism differed, as participants were mixed with those who were thyroidectomized, treated with radioiodine to induce hypothyroidism, and had autoimmune hypothyroidism or pituitary disease. The dose ratio between T3 and T4 varied from 1:4 to 1:20. Moreover, combination therapy also lacks long-term data, including long-term safety. The potential risk with supraphysiological serum fT3 levels during liothyronine and DTE treatment especially warrants caution [63]. European guidelines, in contrast to American, recommend experimental combination therapy in the absence of evidence for 3 months in those with persistent symptoms of hypothyroidism despite adequate dosage with levothyroxine, and thereafter to evaluate [61]. In the absence of prospective long-term follow-up studies with physiological doses of levothyroxine + liothyronine with a positive outcome, monotherapy with levothyroxine remains the standard treatment when hypothyroidism is confirmed [2,51].

A recent blinded prospective study (*n* = 138) investigated whether different doses of levothyroxine aiming for different TSH levels (0.34–2.50, 2.51–5.60, and 5.61–12.0 mIU/L, respectively) affected cognitive symptoms [63]. No difference in cognitive symptoms could be found, and participants could not assess in which group they had participated. The same authors performed a similar study with the same TSH levels, and no differences in weight could be shown [64]. These studies were interesting, but did not address factors such as lipid levels [65], risk for heart failure [66], fatal stroke [67], or the risk for cardiovascular disease and death [68] related to mild hypothyroidism, with TSH >10 mIU/L in different populations [66]. There is some evidence that factors such as hypertension and dyslipidemia improve with levothyroxine therapy, which should be considered when treating younger patients with increased cardiovascular risk. Razvi et al. found that treatment of persistent subclinical hypothyroidism (TSH 5–10 mIU/L) in 40–70 year-old patients was associated with a lower incidence of cardiovascular disease [69].

The association between hypothyroidism and depressive symptoms has been questioned. In manifest hypothyroidism, some depressive symptoms can be relieved with levothyroxine [70]. Blood tests and questionnaires to capture depressive symptoms were examined during a 2 year period in 92,000 middle-aged Koreans [71]. Almost 5% had subclinical hypothyroidism, and 8% developed depressive symptoms. However, there was no difference in developing depressive symptoms in those with subclinical hypothyroidism and those who were euthyroid. Furthermore, in the subgroup with TSH >10 mIU/L (*n* = 326), there were no more depressive symptoms compared to those of the euthyroid controls. These findings imply that depression and hypothyroid symptoms are two different entities.

### 3.4. Could There Be Genetic Causes?

Polymorphisms in deiodinase enzymes converting T4 to T3 or in thyroid hormone transporters may influence the metabolism of thyroid hormones, and potentially patients using levothyroxine replacement. This could explain insufficient well-being in a subgroup of patients. Polymorphism in type II iodothyronine deiodinase (DIO2) was found to be associated with less well-being in hypothyroid individuals treated with levothyroxine, which improved after the addition of triiodothyronine [72]. In contrast, another study could not replicate the result [73]. Moreover, another investigation of patients with polymorphisms in DIO2 found that an increased dose of levothyroxine was required to normalize TSH [74], which could not be confirmed by others [75]. In a small Danish study, 27 out of 45 patients with gene polymorphism in rs225014 (DIO2, Thr92Ala) and/or polymorphism in the thyroid hormone transporter rs17606253 (MCT10) (*n* = 26) preferred the combined treatment more often than patients without such polymorphisms [76]. Future prospective studies may bring more clarity to whether polymorphisms in deiodinase enzymes play a role in a subgroup of patients to guide treatment. Moreover, genome-wide association studies can bring more knowledge on thyroid-hormone metabolism [77].

### 3.5. Subclinical Hypothyroidism in the Elderly

With increasing age, TSH rises in plasma. This phenomenon can in itself be a protective factor [78], associated with longevity [79], and not decreased cognition [80]. A 70-year-old can have a stable physiologically normal TSH of 6 mIU/L, and this does not merit medication [81]. Subclinical hypothyroidism was not found to increase the risk for stroke in patients over 65 years old [67], but was associated with a better outcome [82,83]. In contrast, TSH > 10 mIU/L can be linked with an increased risk of heart failure and other cardiovascular events [67]. This has to be considered, but it has been shown that there are no positive cardiac effects in treating a person >70 years with a TSH < 7 mIU/L [69]. This is controversial, and in recent meta-analysis, subclinical hypothyroidism was associated with increased all-cause mortality in patients >65 years old and a nonsignificant elevated risk of cardiovascular events [84]. However, no randomized prospective study has investigated the effect of levothyroxine therapy on cardiovascular risk in the elderly with subclinical hypothyroidism. Elderly patients are also more vulnerable, often because they are overtreated. Treatment should also take into account the patient´s frailty, especially in the very old [85]. Of >65-year-old patients treated with levothyroxine, 41% had a low TSH (<0.44 mIU/L) [49], which increased the risk for atrial fibrillation, heart failure, and ischemic heart disease [66,67,81,86]. With overtreatment, there was also an increased risk of fractures [84]. The ETA guidelines proposed age-specific TSH ranges to be introduced [3], but there is still no consensus, and individual assessment should be made.

## 4. Conclusions

Most treated patients with hypothyroidism have good well-being. Recent studies have shown that levothyroxine treatment in milder forms rarely affects cognition, weight, or quality of life. Lifelong medication with levothyroxine should normally only be considered in manifest hypothyroidism. In subclinical hypothyroidism with TSH >10 mIU/L, treatment is indicated.

In milder subclinical hypothyroidism, a wait-and-see strategy is advocated to see if normalization occurs. However, individuals with cardiovascular risk and subclinical hypothyroidism may benefit from levothyroxine treatment. Withholding levothyroxine could be advocated in mild forms when clinical improvement does not occur, or if the diagnosis is uncertain but follow-up is required. Persisting symptoms of tiredness and/or weight gain sin someone with normalized thyroid-function test do not require an alternative thyroid medication. Other diagnosis, lifestyle factors, or life events may be the underlying cause.

## Figures and Tables

**Table 1:** Different guidelines on when to initiate levothyroxine treatment.

	TSH Level (mIU/L)	Treatment, Goal TSH (mIU/L)	Addition with DTE	Addition with Liothyronine	First Author (ref. nr.)
ATA	>10	0.5–3.5	No	Not recommended, subgroups may benefit	Jonklass [1]
AACE + ATA	>10 Individualize		No		Garber [2]
BTA	Not specified				Okosieme [18]
ETA	>10 A trial in <65 yo	0.4–2.5	No	Experimentally, dose ratio 13:1–20:1	Pearce [3]
BSEM	>10	No recomm.	No recomm.	No recommendation	Sgarbi [19]
AME		1–3, upper normal in elderly	Divided doses, ratio 10:1–20:1	A trial, dose ratio 10:1–20:1, not in elderly	Guglielmi [20]
Clinical Guidelines	TSH >20			No recommendation	Bekkering [13]

TSH, thyroid stimulating hormone; DTE, desiccated thyroid extract; ATA, American Thyroid Association; AACE, American Association of Clinical Endocrinology; BTA, British Thyroid Association; ETA, European Thyroid Association; BSEM, Brazilian Society of Endocrinology and Metabolism; AME, Italian Association of Clinical Endocrinology.
